# Progress With Livestock Welfare in Extensive Production Systems: Lessons From Australia

**DOI:** 10.3389/fvets.2021.674482

**Published:** 2021-08-06

**Authors:** Peter Andrew Windsor

**Affiliations:** Sydney School of Veterinary Science, The University of Sydney, Camden, NSW, Australia

**Keywords:** climate change, drought, heat, live export, pain management, welfare assessment

## Abstract

The extensive livestock production industries are vital to the national economy of Australia. Continuing improvements to extensively-raised livestock welfare is desirable, necessary and in some situations mandatory, if the social license for animal sourced food and fiber production is to continue sustainably. However, meeting increasingly high welfare standards is challenging. The changing climate in this millennium, has seen the occurrence of two of the most severe drought periods on record in Australia, resulting in complex welfare issues arising from unforeseen disease, trade and environmental catastrophes. The onset of the first drought coincided with an uncontrolled epidemic of ovine paratuberculosis. It ended just prior to a temporary ban on live export of tropical cattle to Indonesia that induced a major market failure and led to severe morbidity and mortality on some beef properties. The second drought period progressed in severity and culminated in the most extreme bushfires recorded, causing unprecedented levels of mortality, morbidity and suffering in farmed animals and wildlife. Temperature extremes have also caused periodic heat-associated or cold-induced hyopthermia losses, requiring increased vigilance and careful management to reduce both temperature-induced stress during transport and the high ovine peri-parturient losses traditionally observed in extensive sheep farming. Several issues remain controversial, including surgical mulesing of wool sheep to manage flystrike, and the continuing live export trade of sheep and cattle. However, in reviewing the increasingly complex welfare challenges for the extensive livestock population industries that are export trade dependent and remain vulnerable to welfare activism, it appears progress has been made. These include development of prescribed livestock welfare Standards and Guidelines and the introduction of the Exporter Supply Chain Assurance System (ESCAS) to address export concerns. Further, the sheep mulesing crisis led to improved producer welfare attitudes and practices, including pain management during aversive husbandry procedures that is now occurring globally. Finally, innovations in animal welfare surveillance and assessment, are additional encouraging signs that suggest improving change management of extensive farm animal welfare is occurring that provides lessons well-beyond Australian shores.

## Introduction

Livestock production accounts for ~40% of agricultural output in developed countries. Advanced genetics, feeding systems, pasture improvements, animal health prevention and controls including improved biosecurity, plus other animal production management technologies, have reduced requirements for livestock by about 20% yet doubled meat production in the last 40 years (1). With global meat production projected to increase another 19% by 2030 (2), improved adoption of emerging “best practice” technologies is required (3). As large ruminant production has now been associated with high outputs of greenhouse gas emissions (GHGe), it has been estimated that improved production efficiencies could potentially assist the global livestock sector to reduce GHGs by as much as 30% (1). Further, recent SARS (including Covid-19) and MERS outbreaks indicate that zoonotic pathogen spillover from livestock production (4) and wild animal populations is occurring, especially where wildlife biodiversity is high and land-use change is occurring (5). Further, there are increasing concerns of emerging food insecurity and safety (6) including antimicrobial resistance (AMR) from misuse of antibiotics, including in food production (7). Finally, with uncontrolled transboundary diseases emerging (e.g., African Swine Fever, Lumpy Skin Disease in SE Asia), it has been suggested that our food system is fragile, requiring radical change to build resilience and ensure our food supply is safer, fairer, and healthier for humans, animals and the environment in the future (8).

However, welfare challenges in livestock production are also considered a major threat to the sustainability of the production of animal-sourced foods (9). In Australia, it has been estimated that 95% of people view farm animal welfare to be a concern and 91% want at least some reform to address this (9). There exists a perceived gap between expectations and regulation, due to an increased focus on animals' level of sentience and related capabilities, with research indicating a fundamental community belief that animals are entitled to the protection of relevant rights and freedoms, closely aligning with activist sentiment (9). The public appears to have an increasing expectation for effective regulation to ensure highly transparent practices in livestock production (9), although achieving this in extensive production systems is challenging. Welfare challenges in extensive husbandry systems in Australia are recognized as mostly associated with: prolonged periods of food and/or water scarcity; climate extremes; high predation risk environments; issues of inadequate surveillance and management of diseases including the monitoring of ill animals with minimal availability of veterinary assistance for parturition, neonatal and other disorders; animal transportation; and biosecurity issues. A number of these challenges are likely to increase in complexity because of climate change. Increased understanding of these challenges and development of potential solutions requires strategic research, particularly for welfare assessment of farm animals in extensive systems and application of innovations that mitigate the impact of these challenges.

Australia has a particular interest in the introduction of new practices to mitigate welfare challenges of extensive livestock production. Extensive livestock production industries are vital to the national economy, and include 26.4 million beef cattle valued at AUD19.6 billion in 2019, and 70.6 million sheep valued at AUD6.6 billion for meat and AUD3.615 billion for wool (in 2019 and 2017, respectively) (10). Due to a number of welfare disaster events in the last two decades, Australian livestock producers are increasingly recognizing that continuing improvements to extensively-raised livestock welfare is desirable, necessary and in some situations mandatory, if the social license for animal sourced food production is to continue sustainably. Australia is a vast country with many marginal soil areas more suitable for extensive livestock grazing than intensive farming. Most sheep occur in the temperate zones of southern Australia, managed extensively in large flocks usually exceeding 3,000 individuals, especially the dominant Merino wool flocks. Some flocks are managed with or adjacent to *Bos taurus* beef cattle, with most enterprises successfully managing this proximity or cohabitation. However, the huge areas of tropical northern Australia are unsuitable for these animals. Livestock located there are mainly *Bos indicus* infused cattle that are raised under very extensive conditions, with climatic extremes, large distances and low management inputs (11). Meeting the increasingly high welfare standards expected is challenging, particularly in northern Australia where animals are rarely mustered more than once annually. Further, as demonstrated in the past two decades, extensive livestock welfare risks are increasing in association with the severity of climate change.

This millenium has seen the onset of two of the most severe drought periods on record in Australia (12–14). That were complicated by welfare issues arising from unforeseen disease, trade and environmental catastrophes. The onset of the first drought coincided with an uncontrolled epidemic of ovine paratuberculosis (15–18). The end of the drought coincided with a temporary ban on live export of tropical cattle to Indonesia that induced market failure, leading to mass morbidity and mortalities on some northern beef properties where extensive management of the animals is sometimes too remote for urgent remediation to prevent welfare issues (19, 20). The second drought period progressed in severity and culminated in the most extreme bushfires recorded, causing high levels of mortality, morbidity and suffering in farmed animals and wildlife (21–26). Temperature extremes also cause periodic heat-associated or cold-induced hyopthermia losses. Increased vigilance and careful management is required to reduce both temperature-induced stress during transport and the high ovine peri-parturient losses traditionally observed in extensive sheep farming. Further, an important issue affecting many extensively grazed properties is control of invasive animal pests causing challenging levels of livestock predation. Several issues remain particularly controversial, including surgical mulesing of wool sheep to manage flystrike, and the continuation of the live export trade of sheep and cattle.

**Figure 1:**
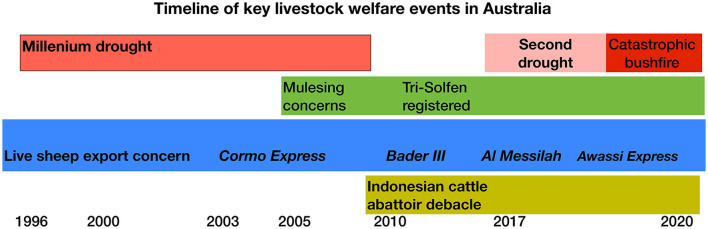
Timeline of key recent livestock adverse welfare events in Australia.

This paper reviews the increasingly complex welfare challenges for the extensive livestock industries that are export trade dependent in Australia and are increasingly vulnerable to both welfare activism and the impacts of a changing climate (Figure 1). The review includes examples of improved producer welfare attitudes and practices through application of research innovations, including pain management during aversive husbandry procedures now occurring globally. Further, it describes new innovations in animal welfare assessment and surveillance and increasing adoption of prescribed welfare standards and guidelines to improve livestock welfare compliance. It concludes that these are encouraging signposts of improved change management of extensive farm animal welfare in Australia that provide lessons relevant to global considerations for the food security system.

## The Changing Climate

Australia's climate has warmed on average by 1.44± 0.24°C since national records began in 1910, with an increase in the frequency of extreme heat events, accompanied by declines of ~16% (April to October) and ~20% (May to July) in rainfall, respectively, since 1970 (12, 13). Similarly, in the southeast of Australia, a decline of ~12% (April to October) rainfall since the late 1990s, with decreasing streamflow at the majority of streamflow gauges across southern Australia since 1975, although rainfall and streamflow have increased across parts of northern Australia since the 1970s. These changes are recognized as increasing the impacts of temperature extremes, causing periodic heat-associated or cold-induced hyopthermia losses, plus increased vigilance and careful management required to reduce both temperature-induced and nutritional-deficiency stress during drought, plus the risk of extreme fire weather and the length of the fire season, across large parts of the country since the 1950s, especially in southern Australia (12, 13). This millenium has seen the onset of two of the most severe drought periods on record in Australia that were complicated by welfare issues arising from unforeseen disease, trade and environmental catastrophes.

The onset of the first and most prolonged so-called “millennium drought” occurred in the southeast of Australia from 1996 to 2010 (14), coinciding with emergence in the late 1990's of an uncontrolled epidemic of ovine paratuberculosis, or Johne's Disease (OJD), causing substantial on farm sheep losses, associated with mortalities exceeding 20% per annum on some farms (15, 16). Necropsy studies identified the mortalities as due to the combined impact of both OJD and drought (17, 18). Paratuberculosis caused about two thirds of the total estimated financial losses (17), with malnutrition accounting for 18% of the annual cost of all deaths among adult sheep (18). This indicated the importance of improving nutritional and disease management practices plus closer flock supervision to reduce the significant biological and financial impacts of OJD on sheep flocks during drought. Drought continues to negatively impact the welfare of both sheep and farming families in Australia, although the severe ovine morbidity associated with OJD and the widespread depression of sheep graziers following precipitous declines in values of affected farms due to OJD in the early millennium years, remains unprecedented (15–17).

In southern and eastern Australia, the recent droughts have been found to be the worst in the past 400 years and expectations are they will become more prevalent in the future (19). The second drought period in eastern Australia was from 2017 to 2020 and led to the entire state of New South Wales (NSW) and more than half of the neighboring state of Queensland to be declared drought-affected. Many described it as the worst drought in living memory, with numerous farmers choosing to cull their cattle due to welfare concerns (20). “Drought Planning” has been intensely promoted by the relevant state agricultural authorities in Australia for over three decades (21, 22). This process includes emergency response planning for “exceptional droughts” that encourage producers to be aware of the need for proactive drought “preparedness, prevention, response, and recovery,” involving systematic: identification of enterprise risks; analysis of climate records; monitoring for trigger points; action planning for when triggers occur; financial analysis and building of cash, feed and water reserves; then regular review and updating of the drought plan. Despite these efforts, it appears that some welfare disasters appear almost impossible to manage effectively. These occur both during and immediately following the drought where there may be precipitous rain causing mass flooding events and even greater losses, both immediate and later disease-induced losses than were associated with the dry period of the drought (23, 24).

Unfortunately, an even more catastrophic post-drought event occurred during this period when in December 2019 through January 2020, eastern Australia experienced its worst ever recorded extreme bushfire season. This culminated in more than 33 people killed, thousands of properties destroyed, at least 18 million hectares of once-green bushland becoming blackened and desolate, with billions of animals dead and injured (25). A new study is currently attempting to improve farmer bushfire preparedness by providing a comprehensive “Livestock Bushfire Preparation and Recovery Manual.” The aim is for farmers to address the physical and financial effects of bushfires that could reduce stock and financial losses incurred by bushfire (26). The study is surveying herd and flock managers affected in the 2019–2020 fire season, assessing animal health and welfare issues, the effects on carcass damage and meat product quality and the financial strain on affected farmers. Further, patterns of burnt areas and livestock losses across landscapes is being assessed to identify a fire risk index that can determine the safest paddocks for stock to be placed in as part of their fire plan (26).

## Livestock Transportation Welfare

The majority of Australian livestock producers and industry personnel, are well aware that to continue their access to domestic and overseas markets, they have important responsibilities for livestock welfare during the large distances required for the transport of animals by road, rail and vehicle onboard ships within and beyond Australia. This has led to the development of the national “Australian Animal Welfare Standards and Guidelines (S&G's) - Land Transport of Livestock” (27) to guide the processes from: the time that animals are mustered and assembled; their handling before and during loading; their journey duration and travel conditions with spelling periods and access to water; then the unloading and holding times. The Land Transport S&G's contain: (i) Objectives describing the intended outcome(s) for each section of the standards; (ii) Standards or minimum requirements that must be met under animal welfare law; and (iii) Guidelines for the recommended practices to achieve desirable animal welfare outcomes to guide and describe higher animal welfare outcomes compared to the minimum requirements of the Standards. This variation in acceptable practices reflects the vast differences in husbandry conditions between different agricultural regions, particularly in the extensive rangelands and tropical northern Australia where livestock farming is more often described as animal “harvesting.” Here, the climatic extremes, large areas and distances within and between holdings (stations or farms) and low management inputs are necessary, ensuring that the extensive tropical cattle industry continues to face significant challenges to assure high standards of animal welfare (11).

However, it is the live export industry (LEI), where more than 2.7 million animals are shipped from Australian ports to nearly 20 countries around the globe annually, that faces the most scrutiny (28), with extensive research indicating that issues posed before, during and after live export results in the cumulative effects of combined stresses on the welfare of the animals (29). This has required the Australian Government assuming animal welfare responsibilities for export abattoirs and the live export trade, despite these issues being difficult to address through regulation and increasingly documented in the media, leading many Australians supporting an end to live export (30). Although welfare incidents in the sheep LEI had occurred sporadically prior to 2003, it was in that year that the “Cormo Express” captured international attention following refusal of entry of the ship and the 57,000 sheep on-board to Saudi Arabia following a claim that some had signs of a vesicular disease, presumably contagious ecthyma or scabby mouth from orf virus infection. The ship spent 2 months moving around the Persian Gulf, with animals exposed to high risk of heat stress, until the animals could be donated to the people of Eritrea (31). Then in 2011, an incident occurred that captured public attention on welfare in the cattle LEI, with the televised filming of disturbing slaughter practices of Australian cattle in Indonesian abattoirs. The exposed animal handling and operational techniques causing pain and injury, led the Federal Government to suspend the trade to Indonesia for a month. The sudden cessation of this AUD1.4 billion LEI of tropical beef cattle caused a precipitous market failure, with the domestic market unable to cope with the sheers numbers of cattle that were now in the supply chain. The impacts of this rapid yet controversial decision created difficult diplomatic, policy and industry issues, that are still debated (32–34) due to the negative impacts on economic returns, community attitudes and international socio-political relations.

The Australian Government in 2011, implemented the Exporter Supply Chain Assurance System (ESCAS) in an attempt to improve control and traceability throughout the LEI supply chain, requiring that transport, handling and slaughter complies with OIE welfare standards (35). However, in August 2012 an Australian ship carrying ~22,000 sheep was blocked from unloading in Kuwait and Bahrain after local authorities also claimed that the animals had orf infection. The sheep had been at sea for 33 days and were left on board for almost a further 2 weeks until they were unloaded in Pakistan, where it was later reported that around 9,000 of the sheep had been killed on suspicion that they were diseased (36). Despite regular vaccination programs, it appears orf infection remains one of the numerous but important threats to the viability of small ruminant LEI's (37). Similar incidents have continued to recur and investigations reveal failures of monitoring and enforcement of ESCAS in destination countries, in both approved and non-approved facilities, most often revealed by the efforts of the various welfare agencies (38). The ongoing concerns have also led to a recent research review of 71 potential animal welfare indicators, categorized as animal, environmental and resource-based measures that would be appropriate for use throughout the LEI for feeder and slaughter livestock species in the 3 LEI sectors: (1) Australian facilities; (2) vessel; and (3) destination country facilities (39). The review identified 38 (sector 1), 35 (sector 2), and 26 (sector 3) measures currently being collected plus 20, 25, and 28 measures that are relevant to each LEI sector (sectors 1, 2, 3, respectively), and that could be developed and integrated into a future benchmarking system (39) should the LEI's continue, presumably as a transition industry until importing countries agree that processed meat is a preferred product to live animals.

## Progress With Pain And Wound Management For Aversive Husbandry Procedures

Perhaps as controversial as the LEI in extensive sheep welfare in Australia, is the “mulesing operation,” a routine procedure with removal of skin from the breech and tail of lambs to create a bare area, providing lifetime prevention against myiasis (flystrike) in susceptible sheep (40). This mostly involves Merino lambs at high risk of the condition because of their breech conformation (wrinkle) that readily retains urine and feces and provides an attractive environment for deposition of the eggs of the sheep blowfly *Lucilia cuprina*. Following hatching, the blowfly larvae burrow deeply into adjacent tissues to the penetrating wounds in afflicted animals, rapidly causing the animal to become moribund because of blowfly strike and if untreated, death. Myiasis remains a serious cause of morbidity and mortality in Australian sheep despite long-term genetic improvement to reduce “blowfly-susceptibility” (41).

Until 2005, mulesing was performed without analgesia, resulting in welfare concerns for the lambs at and following surgery. Then a product designed to be readily used by producers, comprising a “stay and spray” approach for open wounds using a topical anesthetic formulation (TAF) to alleviate pain, plus components to minimize hemorrhage and provide antiseptic cover, was introduced (Tri-Solfen®, Medical Ethics, Australia). On application, it forms a long-lasting biocompatible barrier over the wound, creating its own intrinsic analgesic properties and acting as a slow-release carrier for the actives, including the two local anesthetics, lidocaine hydrochloride (5% w/w) and bupivacaine hydrochloride (0.5% w/w), in addition to the vasoconstrictor adrenaline acid tartrate (0.00451% w/w) and the antiseptic cetrimide (0.5% w/w). The combined synergies create prolonged analgesia extending to at least 24 h and well beyond the expected duration of the actives, plus enhanced healing of open wounds (41, 42). The TAF product has been researched extensively prior to and since it was registered for commercial use in 2012 and has been widely adopted by farmers in Australia, enabling the sale of wool classified as “PR” (pain relief) and improved welfare of sheep susceptible to flystrike during the extended period required until genetic alterations of Australian Merino sheep phenotypes can progress sufficiently to successfully address the risk of myiasis. It is estimated that 6–7 million lambs are treated annually, with well over 100 million sheep having now received treatment since the product was first registered.

This innovation has the potential to complement the various approaches to human wound debridement (43, 44). Further, TAF has been demonstrated to be safe and efficacious in managing pain and improving healing of acute surgical wounds incurred during surgical castration and tail docking of lambs (45, 46), surgical castration and dehorning of calves (47–49), and debridement of hoof lesions in cattle to reduce lameness (50). Effective pain relief at marking has been recognized as important in the northern beef industry. Herd musters commonly occur only annually, resulting in a broad range of ages of calves submitted to dehorning and castration, and variable degrees of restraint stress. Additional findings from recent studies with this product, include confirmation of rapid onset of surgical wound analgesia with positive welfare outcomes for an extended period, improved pain management when used with a non-steroidal anti-inflammatory drug (NSAID, especially meloxicam) or other products for pain relief (51–55), plus on occasions, improved productivity (56). More recently, the TAF has been successfully applied to lesions resulting from viral infection of the mucosa and epidermis, including Foot-and-Mouth Disease (57, 58).

The inclusion of an NSAID in the pain management research in animals is aimed at developing “best practice” options for multimodal pain management, where practical delivery of both the blockage of nociception and amelioration of wound sensitization is achieved. A method for oral delivery of NSAIDs (meloxicam as Ilium Buccalgesic®, Troy Laboratories, Australia) was developed in Australia and has been shown to be efficacious for procedures in both lambs and calves (52, 53). Although the widespread adoption by farmers of the addition of an oral NSAID to surgical procedures in sheep currently remains uncertain, the use of TAF accompanied by intramuscular injections of an NSAID, administered by beef farmers under veterinary advice for castration and dehorning, appears to be rapidly increasing in northern Australia. Recent research has confirmed both efficacy and productivity improvements with this multimodal approach when used in routine husbandry procedures on extensive beef cattle properties (56). In the Australian sheep industry, despite demonstration of the efficacy of an intra-scrotal and tail-docking wound spray of the TAF (45, 46), the convenient use of elastrator bands to cause ischaemic necrosis of the tail and scrotal tissues by producers, remains popular. An instrument recently developed (Numnuts®, Senesino Pty Ltd, Australia) that assists intravenous administration of lignocaine to the neck of the scrotum or tail, prior to application of the band(s), has been shown to reduce pain avoidance behaviors post-procedure (59). As with previous studies with the TAF, use of a multi-modal approach with an NSAID is likely to provide superior pain management.

## Progress With Surveillance, Assessment And New Technologies

With the vast distances occurring on many Australian properties, addressing surveillance challenges for improved welfare in the extensive livestock industries has been recognized as an issue for many years. This is of particular concern with the demands and costs of managing endemic myiasis (*Lucilia cuprina*) and sheep lice (*Bovicola ovis, Lignognathus ovillus* and *Linognathus pedalis*) infestations in southern wool sheep flocks, and cattle tick (*Boophilus microplus*) and buffalo fly (*Haematobia irritans*) in the northern beef cattle herds. For both industries, the impacts of prolonged drought have proven to be extremely challenging, particularly with the recent example of the slow recognition of the moderate to high mortality rates emerging in adult sheep flocks with OJD that were only measurable when wool sheep were mustered for their annual shearing and required on-farm necropsy studies for definitive data (15–18). Similarly, the high background of lamb mortality in many flocks is not as well-recognized by producers as could be expected. This is because in many locations, dead lambs are rarely observed as they may be scavenged by wild canids and in some locations, feral pigs. High lamb mortality potentially reduces profitability and is increasingly perceived as an animal welfare issue for the sheep industry. Yet under extensive sheep production systems, especially in Merino flocks where the disturbance of lambing is to be avoided to minimize mis-mothering, data on mortality rates is usually only available when the flock is mustered for lamb marking and weaning. These interventions usually occurs ~6–8 and 12–14 weeks after the commencement of lambing, respectively. A recent study of producer knowledge of lamb mortality rates, causes of lamb mortality, perceptions and practices that may contribute to lamb deaths, identified that ~50% of producers estimated the mortality rate of lambs between birth and marking was 10%. This compared to published estimates of 20–25% (60). These perceptions impact negatively on the benefits of management strategies, including vaccination. Improved surveillance and extension services are necessary to ensure producers understand the causes of mortality and the key messages required. The generally low predation of live lambs in most cases and the high total costs to farm economics of lamb mortality from failures in disease prevention and management of climatic extremes (60), needs to be addressed.

Similarly, mismanagement of common cattle diseases is potentially a severe and prolonged animal welfare concern (61), with disease prevention almost invariably producing financial benefits that exceed the high costs of disease morbidity and mortality. As in extensive sheep flocks, in very extensive cattle systems, data on reproductive performance and mortality rate is usually only obtained at annual calf marking and weaning. Improving the accuracy of health and productivity records is an important area requiring improvement, with automated technology including drones to regularly visualize the herd, potentially enabling more effective syndromic disease surveillance. This approach remains an inadequately utilized tool that could greatly assist the recognition and diagnosis of welfare issues and disease (61) in extensive production systems. In tropical beef production, there is also the challenge of removing unrequired females from the herd in a suitable condition for sale. This requires that the ovarian function of these females is ablated, usually by spaying to prevent pregnancy and enable fattening (62, 63). The failure of a chemical spaying approach to be effective in the field and the delayed availability of immuno-spaying currently under investigation, has led to continuation of the reasonably common practice of surgical cattle spaying. This is now mostly performed by the Willis Dropped Ovary technique that has a low but recognized mortality rate.

Improving producer knowledge that the potential suffering of disease-affected animals is a welfare issue that needs to be avoided, is important, with timely and humane on-farm euthanasia required when necessary (61). Traditionally, a major driver for improved welfare has been the risk of prosecution via regulatory agencies with statutory responsibilities for ensuring animal welfare compliance. It is anticipated that improved awareness of the Australian Livestock Welfare S&G's could reduce this traditional reliance on regulatory action (27). The traditional reliance on enforcement of current animal welfare legislation in livestock systems in Australia is now being replaced by promotion of self-audits for accreditation schemes. This suggests that objective measurement of animal welfare by appropriate welfare assessment protocols is increasingly important in accompanying efforts to improve surveillance. The welfare relevance of animal health and the relative ease of recording has led to most approaches focusing on clinical measures and physical appearance, with inclusion of behavioral and mental state aspects of welfare suggested as requiring a more comprehensive approach (64). In extensive cow-calf operations, more research is required to develop robust and feasible indicators of positive welfare states for on-farm use. These include objective measures of behavior and the affective state of animals, enabling comparison and contrast of welfare implications of husbandry procedures that are versatile, relevant, reliable, affordable, and broadly acceptable by stakeholders (65). Qualitative Behavioral Assessment (QBA) is an integrated measure that describes behavior as a dynamic, expressive body language, enabling comparative, hypothesis-driven evaluation of various industry-relevant practices. Although most other welfare assessment methods record “problems” such as lameness or injury scores, QBA also captures positive aspects of animal welfare that occur when animals engage with their environment. As QBA is increasingly used in animal welfare assessments in Europe, it may have application in combination with other methods as a welfare assessment tool for the Australian livestock industries (65).

Continuous measurement and monitoring of the behavioral state of animals by using on-animal sensors to identify movements and locations that reflect the well-being of the animals, has potential for extensive livestock systems (66). With increasingly reliable animal welfare measures and decreasing costs of on-animal sensors, technology adoption will very likely increase, particularly if the value proposition for farm businesses and algorithm development, ensures validity and reliability (66). The application of new technologies to improve livestock management systems for improved animal welfare, should complement the learning abilities of the animals (67). Examination of virtual fencing identified that successful learning occurs when the animal perceives cues to be predictable (e.g., an audio warning always precedes a shock) and controllable (e.g., operant response to the audio cue prevents receiving the shock), with an acceptable management also ensuring that welfare is not compromised (67).

## Discussion And Conclusion

Although animal welfare issues facing the extensive southern Australian sheep and beef cattle industries have some similarities to those faced by extensive livestock production industries in many other countries, management of external parasitism from myiasis and lice in wool sheep flocks and the impacts of prolonged drought have proven to be extremely challenging. However, whilst the extensive tropical Australian beef cattle industry in northern Australia is also characterized by climatic extremes and external parasitism from cattle tick and buffalo fly, the large property sizes and distances requiring prolonged transport, plus the necessary low management inputs, ensure the industry still has significant challenges in meeting increasing standards for animal welfare. Issues remain with the mustering and moving of cattle, the timing and frequency of handling; transportation including live export, predation and aversive surgical husbandry procedures (11). With conversion of the northern herd to *Bos indicus* animals better adapted to the northern Australian environment, many of the previous livestock welfare problems have been ameliorated to some degree. Increasing implementation of management changes, including adoption of pain management for surgical procedures, improved planning for extended dry periods and drought, with wider use of supplementary feeding; and broader implementation of vaccination and weaner management programs, suggests dramatic improvement for large numbers of cattle is in progress. Research continues for less-invasive alternatives to cow spaying, and the calf marking procedure could be improved by increasing the adoption of polled genotypes to reduce dehorning plus the earlier castration of males, requiring more frequent mustering of herds.

The LEI's also continue to be problematic. It would appear that patience of many Australians is exhausted with the sheep LEI that sends temperate woolled animals on prolonged sea journeys to countries with harsh tropical environments. This presumably remains an industry in transition. With the exception of variable recipient country slaughter processes and despite some challenges with implementing ESCAS, the issues with the beef LEI, where tropically adapted animals experience far shorter journeys to neighboring tropical countries, appear more defensible. In northern Australia, a considerable number of livestock farming operations are geared for servicing the beef LEI and there is considerable resistance to cessation of this industry. With demand from importing countries likely to increasingly switch to processed meat as a preferred product to live animals, as refrigeration and supermarket sales become more established in regional developing countries, it is envisaged that eventually the northern beef LEI will transition to in-country processing and carcass exports.

Millions of global farm animals experience painful livestock management procedures annually and there is an increased requirement for producers to implement pain management protocols on farms, although in many countries, options for on-farm analgesia are limited (68). Whilst further research is needed on objective measurement of pain in food animals, the use of multimodal analgesia using local anesthetics and particularly TAF, with an NSAID and in particular meloxicam, are currently considered the best options for on-farm analgesia in Australia (44, 69). Further research on pain assessment and amelioration, including applications for inflammatory (57, 58) and neuropathic conditions are necessary to achieve best practice in livestock pain management (68, 69).

There are encouraging signs suggesting improving change management of extensive farm animal welfare is occurring in Australia. These include the adoption of prescribed livestock welfare Standards and Guidelines, introduction of ESCAS to address export concerns (70), improved producer welfare attitudes and practices including pain management during aversive husbandry procedures now occurring globally, plus new innovations in animal welfare surveillance and assessment. With exception of the continuation of the LEI, evidence suggests that a new paradigm has emerged on-farm, capable of sustainably addressing the complex welfare concerns arising in extensive livestock husbandry systems in Australia. Global consumers of extensively-raised livestock products likely need greater awareness of the quality of product raised under improving attitudes and practices of animal welfare on many Australian livestock farms. These lessons may provide valuable insights for producers in and advisors to, the extensive livestock industries in other countries.

## Author Contributions

PW contributed the entirety of this review, although this work was dependent on a number of colleagues and collaborators as per acknowledgments featured as co-authors in the publications cited.

## Conflict of Interest

The author reports no conflict of interest in this work. The extensive studies evaluating Tri-Solfen and other therapies for aversive animal-husbandry interventions that occurred prior to this review paper, were funded by an Australian Research Council Linkage Grant from the Australian government with financial contributions from Medical Ethics Australia and Bayer Animal Health Australia and the author has provided advice to these companies on the international use of pain management products. However, the content in this paper was not influenced by funding from either of these companies, nor did they have a role in the design, decision to publish, or preparation of the manuscript. The handling editor declared a past co-authorship/collaboration with the author.

## Publisher's Note

All claims expressed in this article are solely those of the authors and do not necessarily represent those of their affiliated organizations, or those of the publisher, the editors and the reviewers. Any product that may be evaluated in this article, or claim that may be made by its manufacturer, is not guaranteed or endorsed by the publisher.

## References

[1] FAO. World Livestock: Transforming the Livestock Sector through the Sustainable Development Goals. Rome: FAO (2011). p. xvi–xxxi. Available online at: http://www.fao.org/3/CA1201EN/ca1201en.pdf (Accessed October 20, 2020).

[2] FAO. Mapping supply and demand for animal-source foods to 2030. in Animal Production and Health Working Paper 2. Rome: FAO (2018). Available online at: https://www.fao.org/3/i2425e/i2425e00.htm (Accessed October 20, 2020).

[3] Windsor P Martin S Khounsy S Young J Thomson P Bush. Improved milk production from supplementation of swamp buffalo with molasses nutrient blocks containing 10% urea. Dairy. (2021) 2:90–103. 10.3390/dairy2010009

[4] Becker DJ Washburne AD Faust CL Mordecai EA Plowright RK. The problem of scale in the prediction and management of pathogen spillover. Philos T Roy Soc B. (2019) 374:20190224. 10.1098/rstb.2019.0224 31401958 PMC6711304

[5] Allen T Murray KA Zambrana-Torrelio C Morse SS Rondinini C Di Marco. Global hotspots and correlates of emerging zoonotic diseases. Nat Commun. (2017) 8:1124. 10.1038/s41467-017-00923-8 29066781 PMC5654761

[6] Marchant-Forde JN. The science of animal behavior and welfare: challenges, opportunities and global perspective. Front Vet Sci. (2015) 2:16. 10.3389/fvets.2015.00016 26664945 PMC4672293

[7] Chantziaras I Boyen F Callens B Dewulf J. Correlation between veterinary antimicrobial use and antimicrobial resistance in food-producing animals: a report on seven countries. J Antimicrob Chemoth. (2014) 69:827–34. 10.1093/jac/dkt443 24216767

[8] Marchant-Forde JN Boyle LA. COVID-19 effects on livestock production: a one welfare issue. Front Vet Sci. (2020) 7:585787. 10.3389/fvets.2020.585787 33195613 PMC7554581

[9] Futureye. Australia's Shifting Mindset on Farm Animal Welfare. Windsor (2018). Available online at: https://www.outbreak.gov.au/sites/default/files/documents/farm-animal-welfare.pdf (Accessed February 17, 2021).

[10] ABS. Livestock Products, Australia, December 2020. Australian Bureau of Statistics (2020). Available online at: abs.gov.au (Accessed February 17, 2020).

[11] Petherick JC. Animal welfare issues associated with extensive livestock production: the northern Australian beef cattle industry. App An Behav Sci. (2005) 92:211–34. 10.1016/j.applanim.2005.05.009

[12] BOM. Annual climate statement 2019. Bureau of Meteorology (2019). Available online at: http://www.bom.gov.au/climate/current/annual/aus/2019/ (Accessed February 17, 2021).

[13] BOM. State of the Climate 2020. Bureau of Meteorology (2020). Available online at: http://www.bom.gov.au/state-of-the-climate/ (Accessed February 17, 2021).

[14] BOM. Recent rainfall, drought and southern Australia's long-term rainfall decline. Bureau of Meteorology (2015). Available online at: http://www.bom.gov.au/recentrainfall,droughtandsouthernAustralia'slong-termrainfalldecline (accessed February 17, 2021).

[15] Windsor PA. Paratuberculosis control in sheep and goats. Vet Micro. (2015) 181:161–9. 10.1016/j.vetmic.2015.07.019 26255556

[16] Windsor PA Whittington RJ. Control of ovine paratuberculosis in Australia revisited. Animals. (2020) 10:1623. 10.3390/ani10091623 32927843 PMC7552279

[17] Bush RD Windsor PA Toribio JA. Losses of adult sheep due to ovine Johne's disease in 12 infected flocks over a 3-year period. Aust Vet J. (2006) 84:246–53. 10.1111/j.1751-0813.2006.00001.x 16879127

[18] Bush RD Toribio JA Windsor PA. The impact of malnutrition and other causes of losses of adult sheep in 12 flocks during drought. Aust Vet J. (2006) 84:254–60. 10.1111/j.1751-0813.2006.00002.x 16879129

[19] Doyle K. What you need to know about droughts: why they happen and how they are defined. (2018). Available online at:https://www.abc.net.au/news/2018-08-01/what-you-need-to-know-about-droughts/10051956 (accessed February 17, 2021).

[20] Mao F. Living with Australia's drought: ‘It's cheaper to shoot the cows'. (2018). Available online at: https://www.bbc.com/news/world-australia-45123925 (Accessed February 22, 2021).

[21] Meaker G McCormick L Blackwood I. Drought Planning. Department of Primary Industries, NSW (2007). Available online at:https://www.dpi.nsw.gov.au/climate-and-emergencies/droughthub/information-and-resources/drought-planning (Accessed February 22, 2021).

[22] DPI NSW. Managing farm businesses during drought. Department of Primary Industries NSW. (2018). Available online at: https://www.dpi.nsw.gov.au/climate-and-emergencies/droughthub/information-and-resources (Accessed February 22, 2021).

[23] Mao F. Australian farmers' long road after mass cattle deaths. (2019). Available online at:https://www.bbc.com/news/world-australia-47274662 (Accessed January 22, 2021).

[24] MLA. Flood Recovery. Meat and Livestock Australia (2020). Available online at: https://www.mla.com.au/research-and-development/dealing-with-natural-disasters/flood-recovery (Accessed February 22, 2021).

[25] Wheeling K. Australia's most extreme bushfire season, statistically speaking (2020). Eos. 10.1029/2020EO151949

[26] Hooper C. New study into the effects of bushfires on cattle and sheep health and welfare. (2020). Available online at: https://about.unimelb.edu.au/newsroom/news/2020/july/new-study-into-the-effects-of-bushfires-on-cattle-and-sheep-health-and-welfare (Accessed February 22, 2021).

[27] AHA. Animal Welfare Standards 2020. Animal Health Australia. Animal Welfare Standards Land Transport (Accessed March 01, 2020).

[28] ABC. What would Australia look like without live exports? Australian Broadcasting Corporation News (2018) (Accessed March 01, 2021).

[29] Phillips CJ. ‘The Welfare of Livestock During Sea Transport' in Michael C. Appleby. (eds), Long Distance Transport and Welfare of Farm Animals. CABI. (2008) 137:140. 10.1079/9781845934033.0137

[30] ABC. Vote Compass finds almost two-thirds of Australian voters support a ban on live animal exports (2019). Available online at: https://www.abc.net.au/news/2019-04-26/vote-compass-live-exports-almost-two-thirds-support-ban/11046230 (Accessed March 01, 2021).

[31] SMH. Cormo Express disaster haunts industry. Sydney Morning Herald (2004). Available online at: https://www.smh.com.au/national/cormo-express-disaster-haunts-industry-20041029-gdk0f8.html (Accessed March 01, 2021).

[32] ABC. Politicians link suicides to cattle export ban. Australian Broadcasting Corporation News (2012). Available online at: https://www.abc.net.au/news/2012-09-12/politicians-link-suicides-to-cattle-export-ban/4256526 (Accessed March 01, 2021).

[33] Wagstaff J. Effects of Australian live export cattle ban to Indonesia still felt by beef producers. (2016). The Weekly Times. Available online at: weeklytimesnow.com.au (Accessed February 22, 2021).

[34] Wahlquist C. Australian government's 2011 cattle live export ban was invalid, court rules. (2020). Live exports | The Guardian (Accessed February 22, 2021).

[35] Petrie C. Live export - a chronology. Parliament of Australia (2019). Available online at: https://www.aph.gov.au/About_Parliament/Parliamentary_Departments/Parliamentary_Library/pubs/rp/rp1920/Chronologies/LiveExport (Accessed March 01, 2020)

[36] ABC. Stranded live export sheep unloaded at Kuwait. Australian Broadcasting Corporation News Blog (2012). Available online at: https://www.abc.net.au/news/2012-09-04/stranded-live-export-sheep-unloaded-at-kuwait/4241916 (accessed February 17, 2021).

[37] Windsor PA Nampanya S Tagger A Keonam K Gerasimova M Putthana V. Is orf infection a risk to expanding goat production in developing countries? A case study from Lao PDR. Small Rumin Res. (2017) 154:123–8. 10.1016/j.smallrumres.2017.08.003

[38] RSPCA. Mapping decades of live export disasters. Royal Society for the Protection of Cruelty to Animals, Australia. (2018). Available online at: https://www.rspca.org.au/live-exports-timeline-tragedy (accessed February 17, 2021).

[39] Fleming PA Wickham SL Dunston-Clarke EJ Willis RS Barnes AL Miller DW. Review of livestock welfare indicators relevant for the Australian live export industry. Animals. (2020) 10:1236–26. 10.3390/ani10071236 32708293 PMC7401645

[40] Lomax S Shiel M Windsor PA. Impact of topical anaesthesia on pain alleviation and wound healing in lambs following mulesing. Aust Vet J. (2008) 86:159–69. 10.1111/j.1751-0813.2008.00285.x 18454833

[41] Windsor PA Lomax S. Addressing welfare concerns regarding control of cutaneous myiosis in Australia. Small Rumin Res. (2013) 110:165–9. 10.1016/j.smallrumres.2012.11.027

[42] Lomax S Sheil M Windsor PA. Duration of action of a topical anesthetic formulation for pain management of mulesing in sheep. Aust Vet J. (2013) 91:160–7. 10.1111/avj.12031 23521101

[43] Windsor PA Lomax S White P. Pain management for improved small ruminant welfare. Small Rumin Res. (2016) 142:55–7. 10.1016/j.smallrumres.2016.03.024

[44] Roberts CD Windsor PA. Innovative pain management solutions in animals may provide improved wound pain reduction during debridement in humans: an opinion informed by veterinary literature. Intl Wound J. (2019) 16:968–73. 10.1111/iwj.13129 30938098 PMC7948712

[45] Lomax S Dickson H Sheil M Windsor PA. Topical anaesthesia alleviates the pain of castration and tail docking in lambs. Aust Vet J. (2010) 88:67–74. 10.1111/j.1751-0813.2009.00546.x 20402687

[46] Ferrer LM Lacasta D Ortín A Ramos JJ Tejedor MT Borobia M. Impact of a topical anaesthesia wound management formulation on pain, inflammation and reduction of secondary infections after tail docking in lambs. Animals. (2020) 10:1255. 10.3390/ani10081255 32722010 PMC7459688

[47] Lomax S Windsor PA. Topical anaesthesia mitigates the pain of castration in beef calves. J Anim Sci. (2013) 91:1–8. 10.2527/jas.2012-5984 23965386

[48] Grandin T. Animal welfare and society concerns - finding the missing link. Meat Sci. (2014) 98:461–9. 10.1016/j.meatsci.2014.05.011 24928166

[49] Espinoza C Lomax S Windsor PA. Topical anaesthesia provides pain management for dehorning of calves. J Dairy Sci. (2013) 96:2894–902. 10.3168/jds.2012-5954 23477817

[50] Stilwell GT Ferrador AM Santos S Domingues JM Carolin N. Use of topical local anesthetics to control pain during treatment of hoof lesions in dairy cows. J Dairy Sci. (2019) 102:6383–90. 10.3168/jds.2018-15820 31030913

[51] Paull DR Lee C Colditz IG Atkinson SJ Fisher AD. The effect of a topical Anaesthetic formulation, systemic Flunixin and Carprofen, singly or in combination, on cortisol and Behavioural responses of merino lambs to Mulesing. Aust Vet J. (2007) 85:98–106. 10.1111/j.1751-0813.2007.00115.x 17359309

[52] Small AH Belson S Holm M Colditz IG. Efficacy of a buccal meloxicam formulation for pain relief in merino lambs undergoing knife castration and tail docking in a randomised field trial. Aust Vet J. (2014) 92:381–8. 10.1111/avj.12241 25256843

[53] Small AH Marini D Dyall T Paull D Lee C. A randomised field study evaluating the effectiveness of buccal meloxicam and topical local anaesthetic formulations administered singly or in combination at improving welfare of female Merino lambs undergoing surgical mulesing and hot knife tail docking. Res Vet Sci. (2018) 118:305–11. 10.1016/j.rvsc.2018.03.006 29567597

[54] Van der Saag D Lomax S Windsor PA Taylor C Thomson P Hall E. Effects of topical anaesthesia and buccal meloxicam on average daily gain, behaviour and inflammation of unweaned calves following surgical castration. Animal. (2018) 1:9. 10.1017/S1751731118000216 29477153

[55] Van der Saag D White P Ingram L Manning J Windsor P Thomson P. Effects of topical Anaesthetic and buccal meloxicam treatments on concurrent castration and dehorning of beef calves. Animal. (2018) 8:35. 10.20944/preprints201801.0294.v1 29495653 PMC5867523

[56] Van der Saag D Lomax S Windsor PA Taylor C White PJ. Evaluating treatments with topical anaesthesia and buccal meloxicam for pain and inflammation caused by amputation dehorning of cal- ves. PLoS ONE. (2018) 13:e0198808. 10.1371/journal.pone.0198808 29897950 PMC5999227

[57] Windsor PA MacPhillamy I Khounsy S Young JR Bush RD. Managing welfare and antimicrobial resistance issues in treating Foot-and-Mouth Disease lesions: a new therapeutic approach. VetMed:ResRep. (2020) 11 99–107. 10.2147/VMRR.S273788 33117659 PMC7549654

[58] Lendzele SS Mavoungou JF Burinyuy KA Armel KA Dickmu SJ Young JR. Efficacy of a topical anaesthetic wound treatment formulation for treating Foot-and-mouth disease. A field trial in Cameroon. TBED. (2020) 68, 2531–2542. 10.1111/tbed.13923 33188655 PMC8359326

[59] Small AH Jongman EC Niemeyera D Lee C Colditz IG. Efficacy of precisely injected single local bolus of lignocaine for alleviation of behavioural responses to pain during tail docking and castration of lambs with rubber rings. Res Vet Sc. (2020) 133:210–8. 10.1016/j.rvsc.2020.09.025 33017801

[60] Kopp K Hernandez-Jover M Robertson S Abuelo A Friend M. A survey of New South Wales sheep producer practices and perceptions on lamb mortality and ewe supplementation. Animals. (2020) 10:1586. 10.3390/ani10091586 32899558 PMC7552230

[61] Toaff-Rosenstein R. Chapter 8: Disease and injury: beyond current thinking about top causes of cattle morbidity. In: Tucker CB, editor. Advances in Cattle Welfare. Sawston: Woodhead Publishing (2018). p. 199–226. 10.1016/B978-0-08-100938-3.00007-3

[62] Petherick JC McCosker K Mayer DG Letchford P McGowan M. Evaluation of the impacts of spaying by either the dropped ovary technique or ovariectomy via flank laparotomy on the welfare of Bos indicus beef heifers and cows. J An Sc. (2013) 91:382–94. 10.2527/jas.2012-5164 23048132

[63] Yu A Van der Saag D Letchford P Windsor PA White P. Preliminary investigation to address pain and haemorrhage following the spaying of female cattle. Animals. (2020) 10:249. 10.3390/ani10020249 32033298 PMC7071044

[64] Winkler C. Chapter 3: Assessment of cattle welfare: approaches, goals, and next steps on farms. In: Tucker CB, editor. Advances in Cattle Welfare. Sawston: Woodhead Publishing (2018). p. 55–69.

[65] Fleming PA Clarke T Wickham SL Stockman CA Barnes AL Collins T. The contribution of qualitative behavioural assessment to appraisal of livestock welfare. An Prod Sci. (2016) 56:1569–78. 10.1071/AN15101

[66] Manning J Power D Cosby A. Legal complexities of animal welfare in Australia: do on-animal sensors offer a future option? Animals. (2021) 11:91. 10.3390/ani11010091 33418954 PMC7825130

[67] Lee C Colditz IG Campbell DLM. A framework to assess the impact of new animal management technologies on welfare: a case study of virtual fencing. Front Vet Sci. (2018) 5:187. 10.3389/fvets.2018.00187 30186841 PMC6110809

[68] Kleinhenz MD Viscardi AV Coetzee JF. On-farm pain management of food production animals. App An Sci. (2021) 37:77–87. 10.15232/aas.2020-02106

[69] Stilwell G Windsor P Broom DM. Pain management for ruminants during common farm husbandry procedures. In: Duarte AF Lopes da Costa L, editors. Advances in Animal Health, Medicine and Production. Cham: Springer (2020). p 27–51. 10.1007/978-3-030-61981-7_2

[70] Exporter Supply Chain Assurance System (ESCAS) - Department of Agriculture (Accessed May 10 2021).

