# Corrigendum: Progress With Livestock Welfare in Extensive Production Systems: Lessons From Australia

**DOI:** 10.3389/fvets.2021.827685

**Published:** 2022-01-10

**Authors:** Peter Andrew Windsor

**Affiliations:** Sydney School of Veterinary Science, The University of Sydney, Camden, NSW, Australia

**Keywords:** climate change, drought, heat, live export, pain management, welfare assessment

In the original article, the **Conflict of Interest** statement was incomplete. A corrected **Conflict of Interest** statement appears below.

**Conflict of Interest:** The studies evaluating Tri-Solfen, and other therapies for aversive animal-husbandry interventions that occurred prior to this review paper, were funded by an Australian Research Council Linkage Grant from the Australian government with financial contributions from Animal Ethics Australia and Bayer Animal Health Australia. The author has provided advice to these companies on the international use of pain management products. However, this review was not influenced by funding from either of these companies, nor did they have a role in the design, decision to publish, or preparation of the manuscript.

The handling editor declared a past co-authorship/collaboration with the author.

The authors apologize for this error and state that this does not change the scientific conclusions of the article in any way. The original article has been updated.

## Publisher's Note

All claims expressed in this article are solely those of the authors and do not necessarily represent those of their affiliated organizations, or those of the publisher, the editors and the reviewers. Any product that may be evaluated in this article, or claim that may be made by its manufacturer, is not guaranteed or endorsed by the publisher.

