# Corrigendum: Progress With Livestock Welfare in Extensive Production Systems: Lessons From Australia

**DOI:** 10.3389/fvets.2022.882457

**Published:** 2022-04-06

**Authors:** Peter Andrew Windsor

**Affiliations:** Sydney School of Veterinary Science, The University of Sydney, Camden, NSW, Australia

**Keywords:** climate change, drought, heat, live export, pain management, welfare assessment

In this original article, there was an error. The conflict of interest statement published was incorrect. The corrected conflict of interest statement is below.

The author reports no conflict of interest in this work. The extensive studies evaluating Tri-Solfen and other therapies for aversive animal-husbandry interventions that occurred prior to this review paper, were funded by an Australian Research Council Linkage Grant from the Australian government with financial contributions from Medical Ethics Australia and Bayer Animal Health Australia and the author has provided advice to these companies on the international use of pain management products. However, the content in this paper was not influenced by funding from either of these companies, nor did they have a role in the design, decision to publish, or preparation of the manuscript.

The author apologize for this error and state that this does not change the scientific conclusions of the article in any way. The original article has been updated.

## Publisher's Note

All claims expressed in this article are solely those of the authors and do not necessarily represent those of their affiliated organizations, or those of the publisher, the editors and the reviewers. Any product that may be evaluated in this article, or claim that may be made by its manufacturer, is not guaranteed or endorsed by the publisher.

